# A Swin Transformer-based model for mosquito species identification

**DOI:** 10.1038/s41598-022-21017-6

**Published:** 2022-11-04

**Authors:** De-zhong Zhao, Xin-kai Wang, Teng Zhao, Hu Li, Dan Xing, He-ting Gao, Fan Song, Guo-hua Chen, Chun-xiao Li

**Affiliations:** 1 College of Mechanical and Electrical Engineering, Beijing University of Chemical Technology, Beijing, 100029 China; 2 State Key Laboratory of Pathogen and Biosecurity, Beijing Institute of Microbiology and Epidemiology, Beijing, 100071 China; 3 Department of Entomology and MOA Key Lab of Pest Monitoring and Green Management, College of Plant Protection, China Agricultural University, Beijing, 100193 China

**Keywords:** Classification and taxonomy, Machine learning, Entomology, Invasive species

## Abstract

Mosquito transmit numbers of parasites and pathogens resulting in fatal diseases. Species identification is a prerequisite for effective mosquito control. Existing morphological and molecular classification methods have evitable disadvantages. Here we introduced Deep learning techniques for mosquito species identification. A balanced, high-definition mosquito dataset with 9900 original images covering 17 species was constructed. After three rounds of screening and adjustment-testing (first round among 3 convolutional neural networks and 3 Transformer models, second round among 3 Swin Transformer variants, and third round between 2 images sizes), we proposed the first Swin Transformer-based mosquito species identification model (Swin MSI) with 99.04% accuracy and 99.16% F1-score. By visualizing the identification process, the morphological keys used in Swin MSI were similar but not the same as those used by humans. Swin MSI realized 100% subspecies-level identification in *Culex pipiens* Complex and 96.26% accuracy for novel species categorization. It presents a promising approach for mosquito identification and mosquito borne diseases control.

## Introduction

Mosquitoes belong to Diptera, and transmit a number of parasites and pathogens resulting in hundreds of millions of infections and approximately 750,000 deaths worldwide each year^1^. Therefore, mosquitoes are considered the number one "animal killer" and are among the most medically important insect taxa. Different species of mosquitoes have different habitats, biological habits and pathogen loads. For example, *Anopheles* mosquitos mainly transmit malaria, which caused an estimated 219 million cases globally, and resulted in more than 400,000 deaths every year^2^, *Aedes* mosquitos transmit dengue, which threaten more than 3.9 billion people in over 129 countries with an estimated 96 million symptomatic cases every year^3^, Zika^4^ and chikungunya^5^. *Culex* mosquitos transmit West Nile virus^6^, Japanese encephalitis virus^7^ and lymphatic filariasis^8^. Because specific vaccines or drugs are not available for the majority of mosquito borne diseases, mosquito control is still the main measure used to prevent and control these diseases. Identifying the present mosquito species is a prerequisite and the basis for effectively preventing and controlling mosquito borne diseases. Only by accurately identifying mosquito species can we understand their breeding characteristics and behavioral habits to develop correct prevention strategies and take targeted measures to ensure rapid control^9–11^.

Currently, mosquito species identification methods rely on the morphological characteristics of mosquitos; these methods are time-consuming, laborious and vulnerable to uncertainties associated with genetic variabilities and phenotypic plasticity^12,13^. Even for experienced taxonomists, it is difficult to identify mosquito Complexes with subtle external morphological differences by their external morphological characteristics. For example, subspecies in the *Culex pipiens* Complex can be classified only by the dissected male genitalia^14,15^. In addition, molecular identification methods are effective but expensive and have high technical requirements. In addition, it is difficult to fully meet the rapidly growing demand for rapid and intelligent mosquito species identification using these traditional classification methods.

With the improvements in computing power, the explosive growth of big data and the advancement of machine learning algorithms, deep learning techniques have been rapidly developed and have begun to be applied in image classification tasks. Convolutional neural network (CNN) models with different structures (LeNet^16^, AlexNet^17^, GoogLeNet^18^, VGGNet^19^, ResNet^20^, SqueezeNet^21^, etc.) have been successively applied to perform automatic mosquito recognition using images. However, due to defects associated with the layer structures of these networks, features are easily lost in the computation of each layer, causing gradient disappearances or explosions; thus, these methods cannot effectively capture the relationships between pixel points. The transformer model, which was originally applied in natural language processing research, can solve the above problems through its single-layer structure and multi-head self-attention mechanism. Therefore, attempts have been made to apply transformer models to in computer vision research, and the effects of these models can match or even outperform CNNs^22–24^.

The main contributions of this study are summarized as follows. (1) We aimed to establish the highest-definition and most-balanced mosquito image dataset to date, including 17 species and 3 subspecies, with a total of 9,900 images at an image resolution of 4464 × 2976 pixels. All the classification and identification features in this dataset achieved the discrimination ability of human eyes. (2) The first Swin Transformer-based mosquito species identification (Swin MSI) model was proposed herein, and the species and sex identification accuracies were 99.04% (F1-score 99.16%). (3) In the test set performed in this study, the subspecies and sex identification accuracies of mosquitos in the *Cx. pipiens* Complex, which are morphologically indistinguishable, were both 100%. (4) The Swin MSI model could identify novel mosquitoes that were beyond our dataset with a 96.26% accuracy (F1-score 98.09%) correct genus attribution. (5) As determined by visualizing the identification process, the morphological keys used by the Swin MSI model were similar but not the same as those used by humans.

The Swin MSI model proposed in this study can perform mosquito species identification more accurately than previously established models and could assist taxonomists in identifying mosquito and achieving effective monitoring and prevention of mosquitoes and the associated transmitted diseases.

## Results

### The framework of Swin MSI

**Figure 1:**
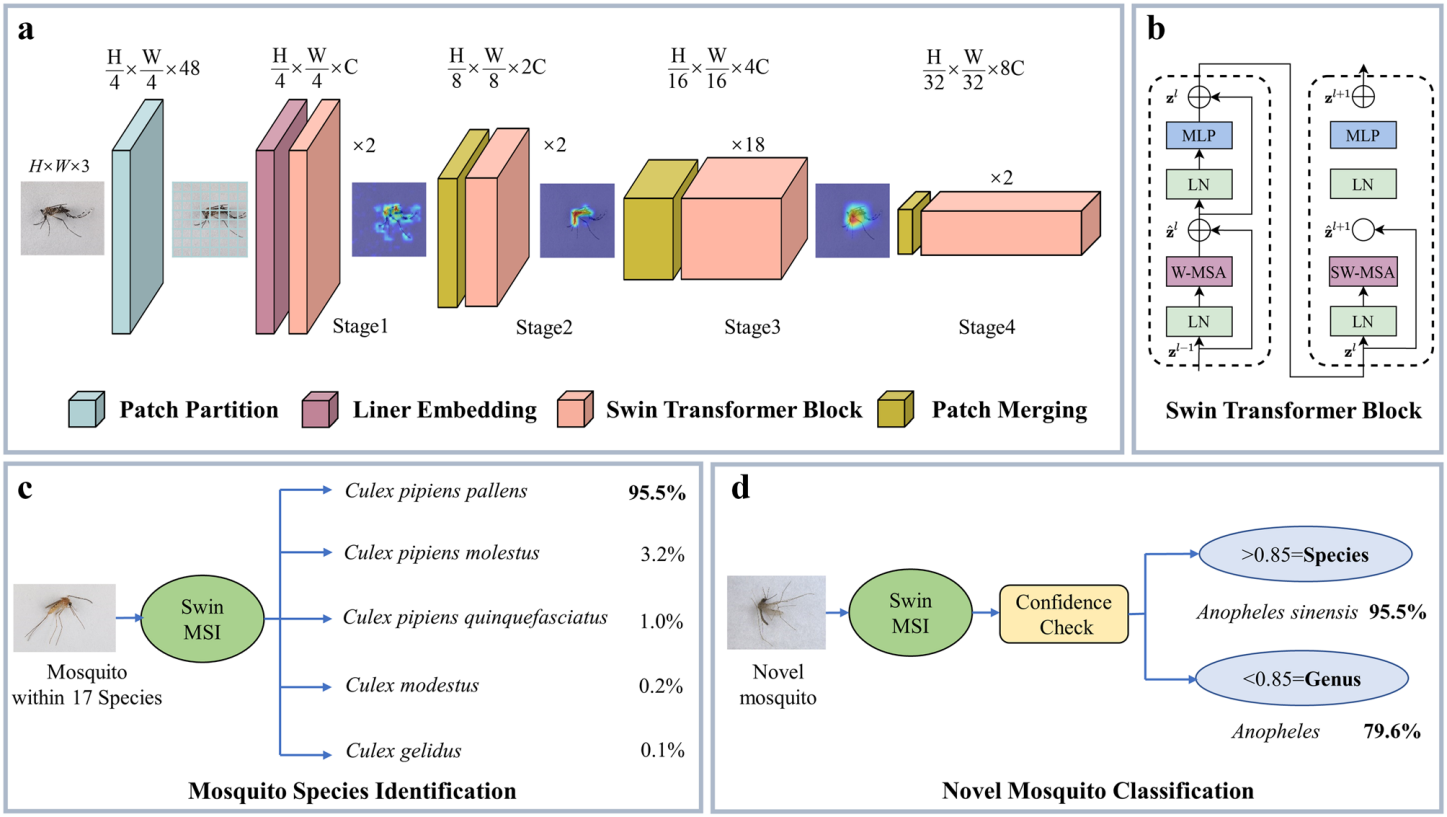
The Framework of Swin MSI. (**a**)The basic architecture for mosquito features extraction and identification. Attention visualization generated by filters at each layer are shown. (**b**) Details for Swin Transformer block. (**c**) For mosquito within our dataset 17 species, output is the top 5 confidence species. (**d**) For mosquito beyond 17 species (defined as novel species), whether the output is a species or a genus is decided after comparing with confidence threshold.

We established the first Swin Transformer-based mosquito species identification (Swin MSI) model, with the help of self-constructed image dataset and multi-adjustment-test. Gradient-weighted class activation mapping was used to visualize the identification process (Fig. 1a). The key Swin Transformer block was described on Fig. 1b. Based on practical needs, Swin MSI was additional designed to identify Culex pipiens Complex on the subspecies level (Fig. 1c) and novel mosquito (which was defined as ones beyond 17 species in our dataset) classification attribution (Fig. 1d). Detailed results are shown in the following sections.

### Mosquito datasets

**Figure 2:**
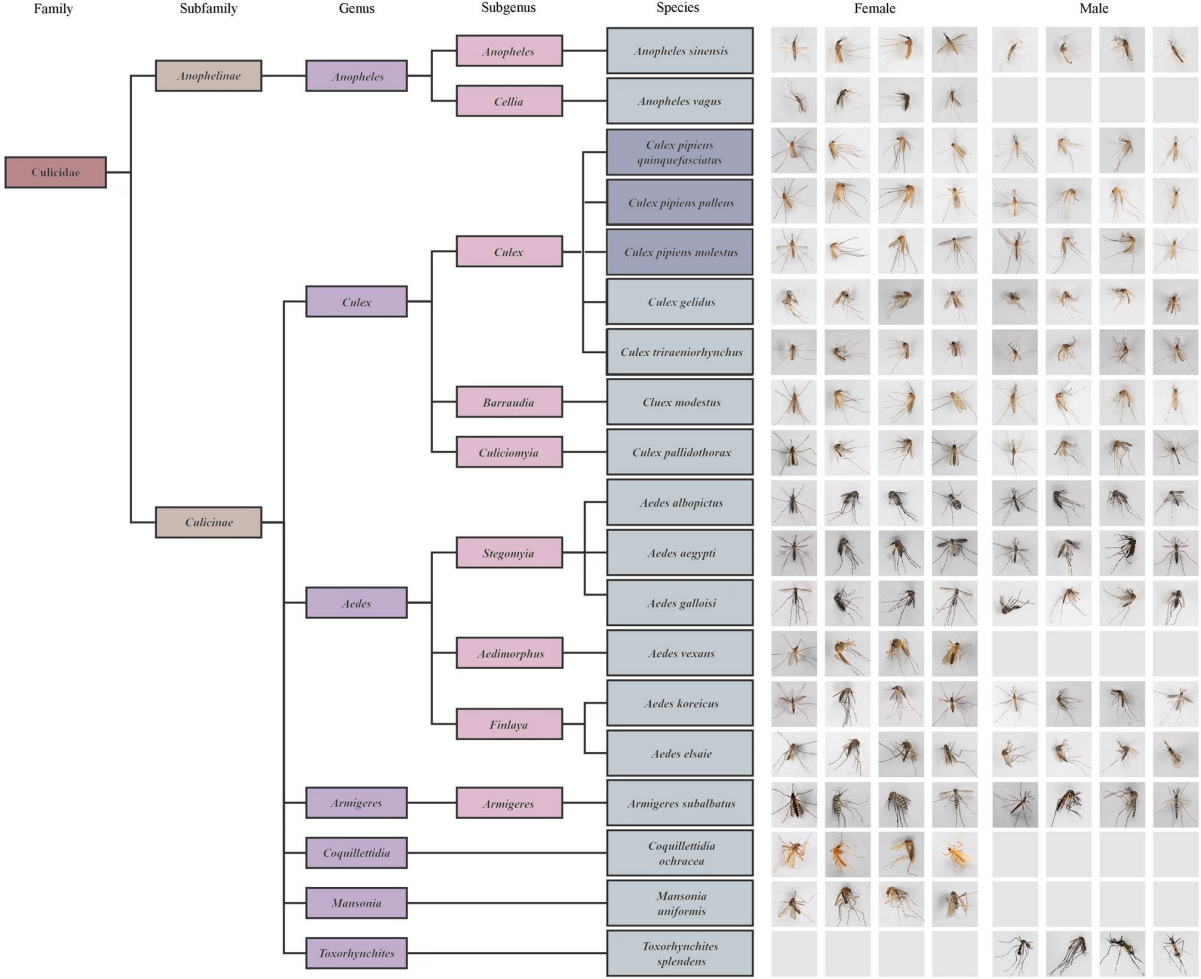
Taxonomic status and index of mosquito species included in this study Both male and female mosquitoes were photographed from different angles such as dorsal, left side, right side, ventral side, etc. Except for 5 species, each mosquito includes 300 images of both sexes, and the resolution of mosquito photos were 4464 × 2976. *Cx. pipiens quinquefasciatus*, *Cx. pipiens pallens*, and *Cx. pipiens molestus* (subspecies level, in dark gray background) were 3 subspecies in *Cx. pipiens* Complex (species level).

We established the highest-definition and most-balanced mosquito image dataset to date. The mosquito image dataset covers 7 genera and 17 species (including 3 morphologically similar subspecies in the *Cx. pipiens* Complex), which covers the most common and important disease-transmitting mosquitoes at the global scale, with a total of 9,900 mosquito images. The image resolution was 4464 × 2976 pixels. The specific taxonomic status and corresponding images are shown in Fig. 2. Due to the limitation of field collection, *Ae. vexans*, *Coquillettidia ochracea*, *Mansonia uniformis*, *An. vagus* and *Toxorhynchites splendens* only have females or only have males. In addition, each mosquito species included 300 images of both sexes, which was large enough and same number for each species, in order to balance the capacity and variety of training sets.

### Workflow for mosquito species identification

**Figure 3:**
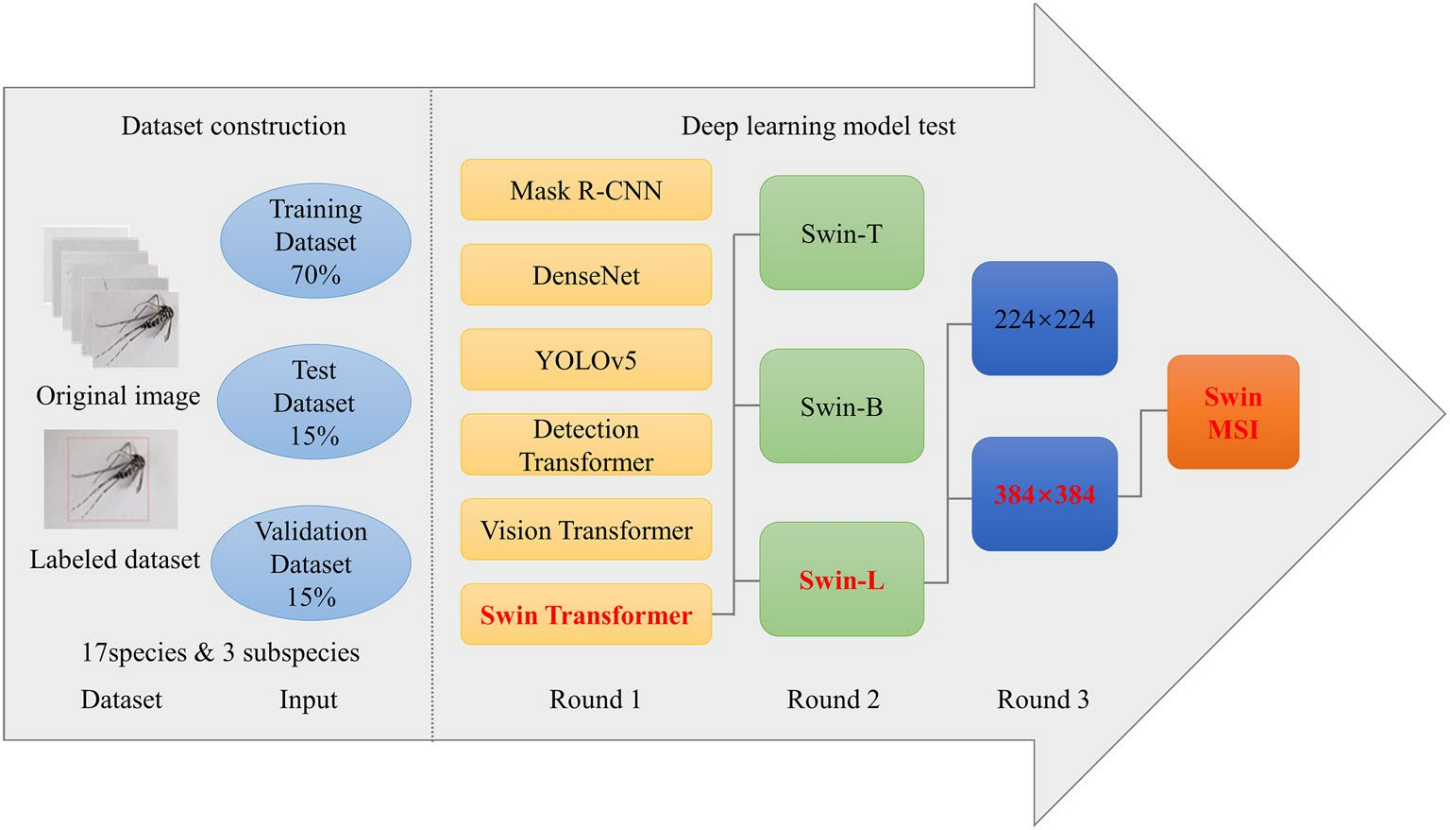
Flowchart of testing deep learning model for intelligent identification of mosquito species.

A three-stage flowchart of building best deep learning model for identification of mosquito species model was adopted (Fig. 3). The first learning stage was conducted by three CNNs (the Mask R-CNN, DenseNet, and YOLOv5) and three transformer models (the Detection Transformer, Vision Transformer, and Swin Transformer). Based on the performance of the first-stage model and the real mosquito labels, the second learning stage involved adjusting the model parameters of the three Swin Transformer variants (T, B, and L) to compare their performances. The third learning stage involved testing the effects of inputting differently sized images (384 × 384 and 224 × 224) to the Swin Transformer-L model; finally, we proposed a deep learning model for mosquito species identification (Swin MSI) to test the recognition effects of different mosquito species. The model was validated on different mosquito species, with a focus on the identification accuracy of three subspecies within the *Cx. pipiens* Complex and the detection effect of novel mosquito species.

### Comparison between the CNN model and Transformer model results (1st round of learning)

**Figure 4:**
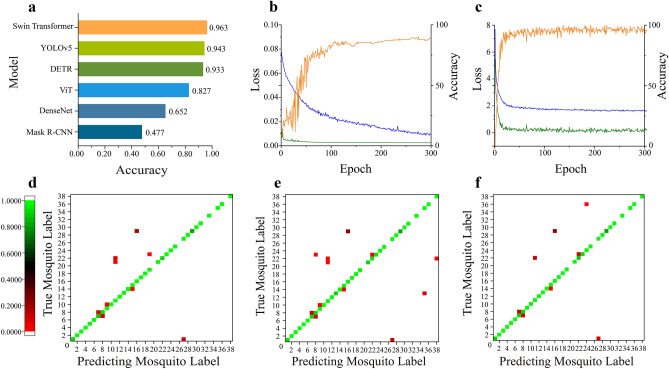
Comparison of mosquito recognition effects of computer vision network models and variants. (**a**) Comparison of mosquito identification accuracy between 3 CNNs and 3 Transformer; (**b**) The best effect CNN (YOLOv5) training set loss curve(blue), validation set loss curve(green) and validation set accuracy curve(orange); (**c**) The best effect Transformer (Swin Transformer) training set loss curve, validation set loss curve and validation set accuracy curve. (**d**) Swin-MSI-T test result confusion matrix; (**e**) Swin-MSI -B test result confusion matrix; (**f**) Swin-MSI -L test result confusion matrix. Confusion matrix of mosquito labels in which odd numbers represent females and even numbers represent males. The small squares in the confusion matrix represent the recognition readiness rate, from red to green, the recognition readiness rate is getting higher and higher *An. sinensis*: 1, 2; *Cx. pipiens quinquefasciatus*: 3, 4; *Cx. pipiens pallens*: 5, 6; *Cx. pipiens molestus*: 7,8 *Cx. modestus*: 9,10; *Ae. albopictus*: 11, 12 *Ae. aegypti*: 13, 14; *Cx. pallidothorax*: 15, 16 *Ae. galloisi*: 17,18 *Ae. vexans*: 19, 20; *Ae. koreicus*: 21, 22 *Armigeres subalbatus*: 23, 24; *Coquillettidia ochracea*: 25, 26 *Cx. gelidus*: 27, 28 *Cx. triraeniorhynchus*: 29, 30 *Mansonia uniformis*: 31, 32 *An. vagus*: 33, 34 *Ae. elsaie*: 35,36 *Toxorhynchites splendens*: 37, 38.

Figure 4a shows the accuracies obtained for the six different computer vision network models tested on the mosquito picture test set. The test results show that the transformer network model had a higher mosquito species discrimination ability than the CNN.

In the CNN training process (applied to YOLOv5), the validation accuracy requires more than 110 epochs to grow to 0.9, and the validation loss requires 110 epochs to drop to a flat interval; in contrast, during the training step, these losses represent a continuously decreasing process. These results indicate that the deep learning model derived based on the Swin Transformer algorithm was able to achieve a higher recognition accuracy in less time than the rapid convergence ability of the CNN during the iterative process (Fig. 4b).

The Swin Transformer model exhibited the highest test accuracy of 96.3%. During the training process, the loss of this model could stabilize after 30 epochs, and its validation accuracy could grow to 0.9 after 20 epochs; during the validation step, the loss can drop to 0.36 after 20 epochs, after which the loss curve fluctuated but did not produce adverse effects (Fig. 4c). Based on the excellent performance of the Swin Transformer model, this model was used as the baseline to carry out the subsequent analyses.

### Swin Transformer model variant adjustment (2nd round of learning)

Following testing performed to clarify the superior performance of the Swin Transformer algorithm, we chose different Drop_path_rate, Embed_dim and Depths parameter settings and labeled the parameter sets as the Swin Transformer-T, Swin Transformer-B, and Swin Transformer-L variants. Drop_path is an efficient regularization method, and an asymmetric Drop_path_rate is beneficial for supervised representation learning when using image classification tasks and Transformer architectures. The Embed_dim parameter represents the image dimensions obtained after the input red–green–blue (RGB) image is calculated by the Swin Transformer block in stage 1. The Depths parameter is the number of Swin Transformer blocks used in the four stages. The parameter information and test results are shown in Table 1. Due to the increase in the Swin Transformer block and Embed_dim parameters in stage 3, the recognition accuracies of the three variants were found to be 95.8%, 96.3%, and 98.2%, Correspondingly, the f1 score were 96.2%, 96.7% and 98.3%; thus, these variants could effectively improve the mosquito species identification ability in a manner similar to the CNN by increasing the number of convolutional channels to extract more features and improve the overall classification ability. In this study, the Swin Transformer-L variant, which exhibited the highest accuracy, was selected as the baseline for the next work.

**Table 1:** Parameters and test accuracy of three variants of Swin Transformer.

Model	Drop_path_rate	Embed_dim	Depths	Batch_size	Accuracy (%)	F1 score (%)
Swin-Transformer-T	0.2	96	[2, 6]	128	95.8	96.2
Swin-Transformer-B	0.5	128	[2, 18]	32	96.3	96.7
Swin-Transformer-L	0.2	192	[2, 18]	8	98.2	98.3

By plotting a confusion matrix of the test set results derived using the three Swin Transformer variants, we clearly obtained the proportion of correct and incorrect identifications in each category to visually reflect the mosquito species discrimination ability (Fig. 4d–f). In the matrix, the darker diagonal colors indicate higher identification rate accuracies of the corresponding mosquito categories. Among them, five mosquito species were missing because the *Ae. vexans*, *Coquillettidia ochracea*, *Mansonia uniformis*, *An. vagus* and *Toxorhynchites splendens* species were represented in the dataset by only females or only males. The confusion matrix shown in Panel C lists the lowest number of mosquito species identification error points and the lowest accuracy level obtained in each category, suggesting that the Swin Transformer-L model has a better classification performance than the Swin Transformer-T and Swin Transformer-B models.

### Effect of the input image size on the discrimination ability (3rd round of learning)

To investigate the relationship between the input image size and mosquito species identification performance, in this study, we conducted a comparison test between input images with sizes of 224 × 224 and 384 × 384, based on the Swin Transformer-L model, and identified 8 categories of mosquito identification accuracy differences. These test results are shown in Table 2. When using an image size of 224 × 224 pixels, the batch_size parameter was set to 16, and when using an image size of 384 × 384 pixels, the batch_size parameter was set to 4; under these conditions, the proportion of utilized video memory accounted for 67%, as shown in Eq. 4, and this was consistent with the description of the relationship between the size of self-attentive operations during the operation of the Swin Transformer model when 384 × 384 pixels images were used. The time required for the Transformer-L model to complete all the training sessions was excessive, reaching 126 h and even exceeding the 124 h required by the YOLOv5 model, which was found to require the highest computation time during the training process in this work. Long-term training process could more fully reflect the performance differences between models. Fortunately and actually, the response speed of the model will not be affected by the training time. Compared to the accuracy of 98.2% obtained for 224 × 224 inputs, the 384 × 384 input image size derived based on the Swin Transformer-L model provided a higher mosquito species identification accuracy of 99.04%, representing an improvement of 0.84%.

1 $$\Omega ({\text{W}} - {\text{MSA}}) = 4{\text{HWC}}^{2} + 2{\text{M}}^{2} {\text{HWC}}$$

**Table 2:** Comparison of recognition accuracy for different input image sizes.

Mosquito label	224 × 224		384 × 384	
	Acc (%)	F1 (%)	Acc (%)	F1 (%)
*An. sinenis female*	91.11	95.35	86.67	92.86
*Cx. pipiens molestus female*	91.11	95.35	100	100
*Cx. pipiens molestus male*	91.11	95.35	100	100
*Ae. aegypti male*	95.56	97.73	97.78	98.88
*Ae. koreicus male*	86.67	92.86	91.11	95.35
*Armigeres subalbatus female*	91.11	95.35	95.56	97.73
*Cx. triraeniorhynchus female*	80.00	88.89	95.56	97.73
*Ae. elsaie male*	95.56	97.73	100	100

Mosquito species with same recognition accuracy between 2 input image sizes were not listed in the table. Acc was short for accuracy and F1 was shoret for f1-score.

### Visualizing and understanding the Swin MSI models

**Figure 5:**
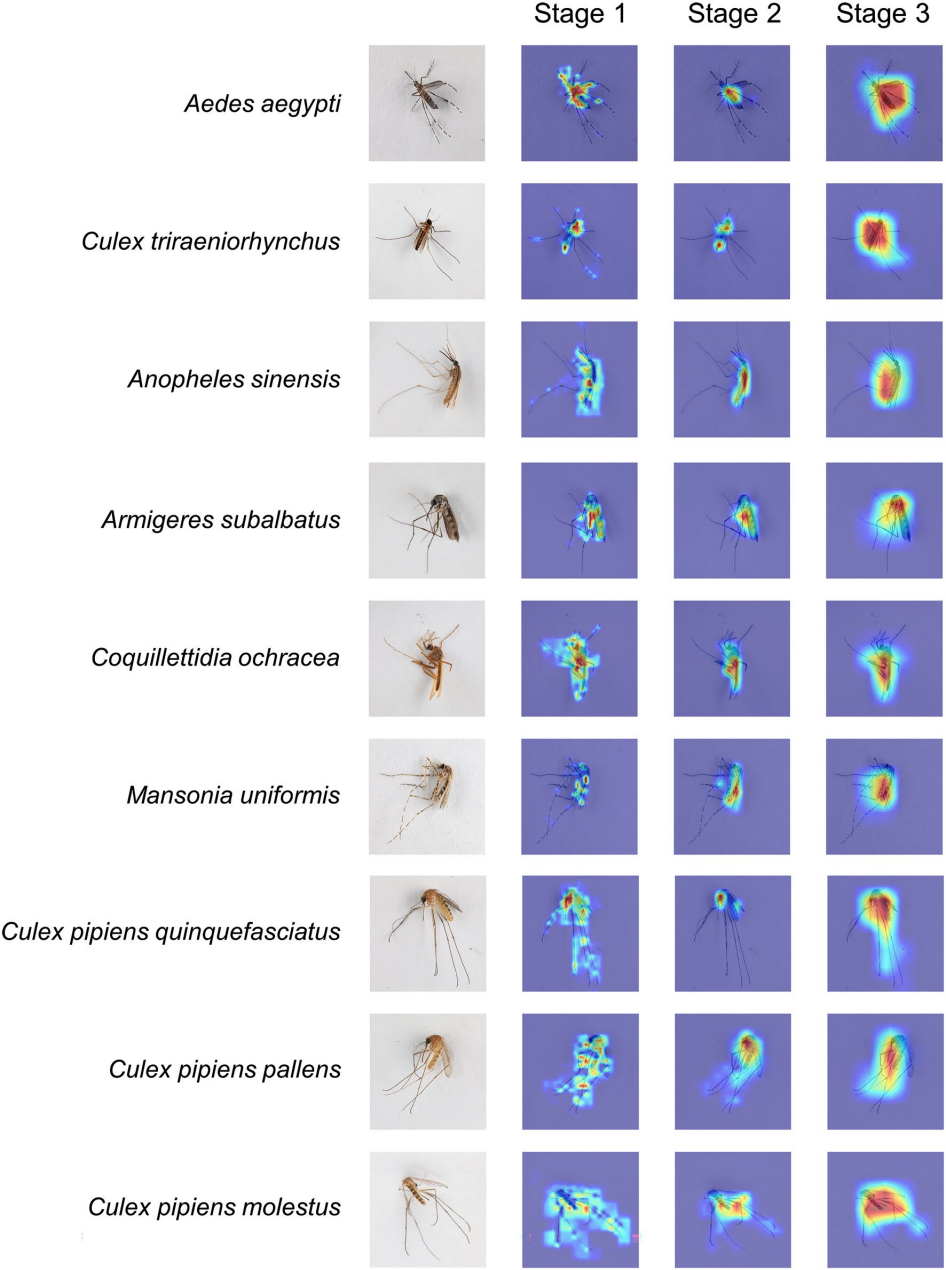
Attention visualization of representative mosquitoes of the genera *Ae.*, *Cx.*, *An.*, *Armigeres*, *Coquillettidia* and *Mansonia*. This is a visualization for identifying the regions in the image that can explain the classification progress. Images of *Toxorhynchites* contain only males, with obvious differences in morphological characteristics, are not shown.

To investigate the differences in the attentional features utilized by the Swin MSI and taxonomists for mosquito species identification, we applied the Grad-CAM method to visualize the Swin MSI attentional areas on mosquitoes at different stages. Because the Swin Transformer has different attentional ranges among its multi-head self-attention steps in different stages, different attentional weights can be found on different mosquito positions. In stage 1, the feature dimension of each patch was 4 × 4 × C, thus enabling the Swin Transformer's multi-head self-attention mechanism to give more attention to the detailed parts of the mosquitoes, such as their legs, wings, antennae, and pronota. In stage 2, the feature dimension of each patch was 8 × 8 × 2C, enabling the Swin Transformer's multi-head self-attention mechanism to focus on the bodies of the mosquitoes, such as their heads, thoraces, and abdomens. In stage 3, when the feature dimension of each patch was 16 × 16 × 4C, the Swin Transformer's multi-head self-attention mechanism could focus on most regions of the mosquito, thus forming a global overall attention mechanism for each mosquito (Fig. 5). This attentional focus process is essentially the same as the process used by taxonomists when classifying mosquito morphology, changing from details to localities to the whole mosquito.

*Ae. aegypti* is widely distributed in tropical and subtropical regions around the world and transmits Zika, dengue and yellow fever. A pair of long-stalked sickle-shaped white spots on both shoulder sides of the mesoscutum, with a pair of longitudinal stripes running through the whole mesotergum, is the most important morphological identification feature of this species. This feature was the deepest section in the attention visualization, indicating that the Swin MSI model also recognized it as the principal distinguishing feature. In addition, the abdominal tergum of *A. aegypti* is black and segments II-VII have lateral silvery white spots and basal white bands; the model also focused on these areas.

*Cx. triraeniorhynchus* is the main vector of Japanese encephalitis; this mosquito has a small body size, a distinctive white ring on the proboscis (its most distinctive morphological feature), and a peppery color on its whole body. Similarly, the model constructed herein focused on both the head and abdominal regions of this species.

*An. sinensis* is the top vector of malaria in China and has no more than three white spots on its anterior wing margin and a distinct white spot on its marginal V5.2 fringe; this feature was observed in Stage 2, at which time the modelstrongly focused on the corresponding area.

The most obvious feature of *Armigeres subalbatus* is the lateral flattening and slightly downward curving of its proboscis; the observation of the attention visualization revealed that the constructed model focused on these regions from Stage 1 to Stage 3. The mesoscutum and abdominal tergum were not critical and were less important for identification than the proboscis, and the attention visualization results correspondingly show that the neural network focused less on these features.

*Coquillettidia ochracea* belongs to the *Coquillettidia* genus and is golden yellow all over its body, with the most pronounced abdomen among the analyzed species. The model showed a consistent morphological taxonomic focus on the abdomen of this species.

*Mansonia uniformis* is a vector of Malayan filariasis. The abdominal tergum of this species is dark brown, and its abdominal segments II-VII have yellow terminal bands and lateral white spots, which are more obvious than the dark brown feature on proboscis. Through the attention visualization, we determined that the Swin MSI model was more concerned with the abdominal region features than with the proboscis features.

### Subspecies-level identification tests of mosquitos in the *Culex pipiens* Complex

Fine-grained image classification has been the focus of extensive research in the field of computer vision^25,26^. Based on the test set (containing 270 images) constructed herein for three subspecies of the *Cx. pipiens* Complex, the subspecies and sex identification accuracies were 100% when the Swin MSI model was used.

The morphological characteristics of *Cx. pipiens quinquefasciatus*, *Cx. pipiens pallens*, and *Cx. pipiens molestus* within the *Cx. pipiens* Complex are almost indistinguishable, but their host preferences, self-fertility properties, breeding environments, and stagnation overwintering strategies are very different^27^. Among the existing features available for morphological classification, the stripes on the abdominal tergum of *Cx. pipiens quinquefasciatus* are usually inverted triangles and are not connected with the pleurosternums, while those of *Cx. pipiens pallens* are rectangular and are connected with the pleurosternums. *Cx. pipiens molestus* is morphologically more similar to *Cx. pipiens pallens* as an ecological subspecies of the *Cx. pipiens* Complex. However, taxonomists do not recommend using the unstable feature mentioned above as the main taxonomic feature for differentiation. By analyzing the attention visualization results of these three subspecies (last three rows on Fig. 5), we found that the neural networks of *Cx. pipiens quinquefasciatus*, *Cx. pipiens pallens*, and *Cx. pipiens molestus* still focused on the abdominal regions, as shown in dark red. The area of focus of these neural networks differ from that of the human eye, and the results of this study suggest that the Swin MSI model can detect finely granular features among these three mosquito subspecies that are indistinguishable to the naked human eye.

### Novel mosquito classification attribution

After we performed a confidence check on the successfully identified mosquito images in the dataset, the lowest confidence value was found to be 85%. A higher confidence threshold mean stricter evaluation criteria, which can better reflect the powerful performance of the model. Therefore, 0.85 was set as the confidence threshold when judging novel mosquitoes. When identifying 10 unknown mosquito species, the highest derived species confidence level was below 85%; when the results were output to the genus level (Fig. 1d), the average probability of obtaining a correct judgment was 96.26%accuracy and 98.09% F1-score (Table 3). The images tested as novel *Ae., Cx.* and *An.* mosquito were from Minakshi and Couret et al.^28,29^.

**Table 3:** Probability of correct attribution of novel species.

Novel Species	Acc (%)	F1 (%)	Tested Image numbers
*Ae. infirmatus*	80.00	88.89	10
*Ae. taeniorhynchus*	80.00	88.89	10
*Cx. coronator*	70.00	82.35	10
*Cx. nigripalpus*	90.00	94.74	10
*An. albimanus*	98.44	99.21	64
*An. arabiensis*	95.85	99.20	193
*An. atroparvus*	100	100	36
*An. coluzzi*	95.95	97.93	74
*An. farauti*	100	100	57
*An. freeborni*	98.97	99.48	97

Acc was short for accuracy and F1 was shoret for f1-score.

## Discussion

In a previous study by Pataki et al.^30^, the highest mosquito species identification accuracy was 96%; this value was obtained with ResNet based on a public pest vector monitoring dataset (containing 6195 *Ae. albopictus* images out of 7686 images, accounting for 80.6%). In Motta et al.'s study^31^, a dataset of 4056 adult mosquito images of three species (*A. albopictus*, *A. aegypti*, and *Cx. pipiens quinquefasciatus*) was constructed and trained using the LeNet, AlexNet, and GoogleNet CNNs, and the test results showed that the best precision was obtained with GoogleNet at 76.2%. Park et al. constructed a dataset containing 3578 images of eight mosquito species, and classification accuracies above 97% was achieved using VGG-16, ResNet-50, and SqueezeNet. Couret et al. constructed a dataset containing 14 species of mosquitoes, mainly within the genus *An.*, for a total of 1709 images; using this dataset, a species identification accuracy of 96.96% and a sex prediction accuracy of 98.48% were achieved using the best-configured DenseNet-201 model.

Obviously, previous mosquito recognition studies used computer vision techniques mostly with ResNet, DenseNet and other classical CNNs. This study is the first to apply the recently emerged Transformer model^32^ for mosquito species identification. CNNs complete the whole training process by using stacked convolutional layers and pooling layers to produce layer-level feature representations of different sizes and perceive image features on various scales. However, the structural design of a CNN itself focuses primarily on the extraction effects of local features and tends to ignore the connections among different elements in an image. The Swin MSI model proposed in this study combines the advantages of both the Transformer and CNN models. The architecture of the constructed model works similarly to CNNs by stacking Swin-transformer blocks and continuously increasing the patch dimensions and sizes; in addition, the use of a self-attentive mechanism based on moving windows effectively reduces the number of parameters generated during the computation process. By dividing the feature map of size into nonoverlapping windows of size, the computation is linear in the square of the window size, thus solving the problem of the high computational complexity of traditional Transformer models. The Swin MSI model allows the new patches to expand in size through a merging process, thus playing the same role as the sensory field in a CNN. In the initial stage, only local features such as antennae, mesoterga, legs or wings can be sensed, but in the large-sized patches formed after several patch-merging steps, global features such as heads, thoraces and abdomens can be sensed, and focus on the mosquitoes overall can eventually be obtained.

The most critical issue in any computer vision detection task is the manual establishment of image annotations (professional mosquito classification staff are required to perform these annotations)^33,34^. Therefore, obtaining a complete and clear dataset for accurate predictions can save considerable time and costs. Except for a study on the *An. gambiae* Complex group, which involving a subspecies-level analysis, previous mosquito classification studies have mostly focused on species with obvious biological taxonomic characteristics, such as *A. aegypti and A. albopictus*.^31^ Park et al. found that some mutilated field-collected mosquitoes prevented the neural network from being able to capture key features, thus causing classification errors^30^. In addition, imbalances in the number of different mosquito species cause neural networks to tend to reduce the recognition quality of minority categories to maximize the overall recognition rate^35^. Learning from previous experiences, the mosquito images collected in this study contain a large number of critical morphological feature, species and sex details and form the clearest and most balanced professional mosquito database thus far; this database ensures the optimal performance of the constructed Swin-MSI model in mosquito species identification tasks.

We found that the higher the resolution of the mosquito image training set was, the better the recognition effect was. The mosquito species identification accuracy was improved by 0.84%, to 99.04%, when the input image size was set to 384 × 384 compared to 224 × 224, other related research results confirm the superiority of large-size image input^36^. Currently, the efficiencies of computer vision network models are limited by the abilities of hardware devices. Although we made some adjustments, including adjustments to the number of model parameters, the constructed model still required substantial computational times on high-performance hardware devices, such as the graphics processing unit (GPU) to perform the data computations and the central processing unit (CPU) to make resource calls. The video memory of the GPU determines the amount of data that can be computed in parallel, which directly affects the training time. Our mosquito dataset had a resolution of 4464 × 2976 pixels and had taken the appropriate redundancy of the image sizes into account. In the future, with the improvements in hardware computing power and adjustments to the patch-merging process to optimize the Swin MSI model structure, the advantages of high-resolution datasets will be maximized, thus leading to even higher accuracies.

In this study, we attempted to combine the experience of professional taxonomists with the perspective of artificial intelligence to determine whether the Swin Transformer model uses similar morphological keys as those used by human experts to classify mosquito species and subspecies. The evolutionary statuses of subspecies within mosquito Complexes are similar, and subspecies within the same Complex have subtle morphological characteristic differences. Unlike the *An. gambiae* Complex, which is mainly found in limited geographic areas^37^, the *Cx. pipiens* Complex group targeted in this study is widely distributed worldwide^38^, and their corresponding blood-sucking host preferences, self-fertility processes, breeding environments, and stagnant overwintering processes are significantly different. Thanks to the high-resolution dataset containing complete morphological characteristic information, 100% subspecies and sex identification accuracies were achieved at the subspecies level for the *Cx. pipiens* Complex. The visualization results suggested that the morphological keys used by the Swin MSI model were similar but not the same as those used by humans. The identification features derived at certain pixel-level details included mosquito heads, thoraces, abdomens, dorsal plates, and legs; these features may serve as useful supplements to traditional taxonomic features and provide a reference with which professional taxonomists can explore the subspecies-level morphological features of mosquitoes in depth.

Although the dataset established in this study already includes 17 species (including 3 subspecies) of mosquitoes, the need to identify novel mosquitoes is common and inevitable in realistic practical applications. The highest species confidence level achieved with the existing Swin MSI model was below 85%, and the correct attribution probability of the model reached 96.3%. This is a stop-gap measure under existing resource (prioritizing coverage of the most dangerous and common mosquitoes), but it is sufficient to help inexperienced personnel on site identify dangerous mosquitoes. By expanding the number of species represented in specific area mosquito dataset and optimizing the model structure and parameters, the species confidence threshold can be continuously increased, leading to higher attribution accuracies for novel mosquitoes. More developed version for different countries and regions to cover their local mosquito species will be move forward in the future. The Swin MSI model can help mosquito taxonomists quickly distinguish new species from a large number of specimens identified in field biodiversity surveys^39^. The model also has a high identification accuracy for damaged mosquito samples collected in the field, as long as the training set contained sufficient recognition features, which makes mosquito surveillance tasks more effective and efficient^40^.

In summary, the first transformer-based mosquito species identification model was proposed in this study. With the help of a self-constructed high-precision mosquito image dataset, the Swin MSI model achieved a species recognition rate greater than 99%. The identification effect is also tested and discussed at various input image sizes, at the subspecies level (at which the morphological features of mosquitos are difficult to distinguish), and for novel species. The excellent performance of the Swin MSI model makes it an accurate and efficient technical tool that will help taxonomists quickly identify mosquito species and contribute to the control of mosquito borne diseases.

## Methods

### Ethic statements

The study was approved by Institutional Animal Care and Use Committee of Beijing Institute of Microbiology and Epidemiology (IACUC-IME-2021-021). The mosquito field sampling was conducted without harming any other animal species.

### Mosquito datasets

In this work, mosquitoes were identified by taxonomists using morphological indicators identified through a standard stereomicroscope. All mosquitoes were killed by anesthesia, and pictures were taken of the mosquitos in the form of natural death. We built a macro-photography platform of mosquito images using of a Canon electro-optical system (EOS) 5D Mark IV digital camera with a Laowa 100-mm F2.8 Macro 2X lens and a Kuangren KR-888 macro flash to capture the dorsal, ventral, left, and right sides of each mosquito, covering the key identification features. Three to five images were taken of each mosquito. the camera and flash parameters were set to ISO400, the shutter speed was set to 1/100 s, the aperture was set to 22, and the flash index was set to 1/16.

### Data division, preprocessing and augmentation steps

The captured mosquito images were labeled using LabelImg software to generate corresponding .txt files containing category, sex, and location coordinate information. A total of 9900 images captured of 17 species(and 3 subspecies) were used according to the principle of 50/50 males and females (since identifying features of males and females were different, each species included 300 images of both sexes. If one sex was missing from the samples, the number of mosquitoes extracted from that species was halved). The images were partitioned randomly into training, validation, and test sets comprising 6930 (70%), 1485 (15%), and 1485 (15%) images in the dataset, respectively. To prevent underfitting, reduce the probability of overfitting, and prevent classification imbalances resulting from unbalanced category shares, we applied 24 rotations with 15-degree increments to substantially increase the size and diversity of the training image set by using rotations, flips, and resizing methods.

### Comparison of the CNN models and transformer model test results

Six computer vision models (the Mask R-CNN, DenseNet, YOLOv5, Swin Transformer, Detection Transformer and Vision Transformer models) were first selected for testing (Table 4)^22–24,41,42^. The Mask R-CNN model uses a feature pyramid network (FPN) architecture to enhance its multiscale feature extraction capability^41^. DenseNet further mitigates the gradient disappearance problem by establishing connections among different layers^42^. YOLOv5 surpasses various previous versions of the YOLO model with a small number of parameters and a strong rapid deployment advantage. Detection Transformer used a CNN to extract features and then used a transformer to perform identification, which truly achieves end-to-end detection with less prior. Dosovitskiy proposed a pure transformer, namely, the Vision Transformer (ViT), to serialize images and applied the transformer to image classification tasks with good results. The Swin Transformer uses a sliding window and introduces a concept similar to the expanded field of perception used in CNNs. The above six models are excellent representatives of CNNs and transformers and have achieved high accuracies when analyzing datasets such as ImageNet in various application scenarios.

**Table 4:** Parameter settings of the six computer vision models.

Model	Numbers of epochs	Learning rate	Batch-size
Detection Transformer	300	0.0001	2
Vision Transformer	500	0.03	512
Swin Transformer	300	0.0005	128
YOLOv5	300	0.001	16
Mask R-CNN	160	0.001	1
DenseNet	300	0.1	16

### MSI model design

By screening the six models described above, the Swin Transformer algorithm model was found to have the best performance in the first testing phase and was ultimately selected for further analysis. The parameter configuration was further adjusted to test the effects of the variants (T, B and L) and input image sizes (384 × 384 and 224 × 224) on the species recognition accuracy. The specific steps will be further explained in the following section.

### Swin MSI framework

The Swin MSI model splits the preprocessed mosquito image into many nonoverlapping patches using a patch-splitting model. The number of patches is $\frac{H}{4} \times \frac{W}{4}$, each patch is considered a token with dimensions of 4 × 4, and the features of each patch are set as a series of pixel values in the preprocessed mosquito image; thus, the number of features in each patch is 4 × 4 × 3 = 48. The linear embedding layer is then applied to the segmented patches to project them to the set dimensions (C).

The computation results of stage 1 were combined with 2 × 2 similarly sized patches by patch merging, the size was increased to 8 × 8, and the number of patches became $\frac{H}{8} \times \frac{W}{8}$; these patches were then used as the input in stage 2. After repeating the self-attention calculation of the Swin Transformer block, the output of stage 2 was obtained, and the calculations were continued in two stages (denoted as stage 3 and stage 4) until the patch size reached 32 × 32. At this time, the number of patches was $\frac{H}{32} \times \frac{W}{32}$, the dimension for 8C. In the process of moving the window, half of the patches were moved each time to ensure that no features were lost in the process. In this work, the input image sizes were set to 224 × 224 and 384 × 384 pixels, and the final patch numbers used in each window were set to 7 × 7 and 12 × 12.

### Shifted windows multihead self-attention

The Swin Transformer block consists of two consecutive Swin Transformer modules: multi-head self-attentive modules with a regular window configuration (W-MSA) and moving window configuration (SW-MSA). A layer normalization processing module (LN ()), a multilayer perceptron (MLP ()), the output feature of the SW-MSA module ($\mathop {Z^{L} }\limits^{ \wedge }$), and the output feature of the MLP features ($Z^{L}$) were also established.

The computation in the continuous Swin Transformer block can be expressed by Eq. (1) as follows:

2 $$\begin{gathered} \mathop {\text{Z}}\limits^{{ \wedge }{\text{l}}} = {\text{W - MSA(LN(Z}}^{{{\text{L}} - {1}}} {))} + {\text{Z}}^{{{\text{L}} - {1}}} {,} \hfill \\ {\text{Z}}^{{\text{l}}} = {\text{MLP(LN(}}\mathop {\text{Z}}\limits^{{ \wedge }{\text{l}}} {))} + \mathop {\text{Z}}\limits^{{ \wedge }{\text{l}}} {,} \hfill \\ {\hat{\text{{Z}}}}^{{{\text{l}} + 1}} = {\text{SW - MSA(LN(Z}}^{{\text{l}}} {))} + {\text{Z}}^{{\text{l}}} {,} \hfill \\ {\text{Z}}^{{{\text{l}} + {1}}} ={\text{MLP}}\left( {{\text{LN}}\left( {\hat{{\text{ {Z}}}}^{{{\text{l}} + 1}} } \right)} \right) + {\hat{\text{ {Z}}}}^{{{\text{l}} + 1}} \end{gathered}$$

The SW-MSA module derived for the nonoverlapping window X can be expressed by Eq. (2) as follows:

3 $$\begin{gathered} MultiHead(Q,K,V) = Concat(head_{1}, \ldots ,head_{h})W^{O} \hfill \\ where\;head_{i} = Attention(QW_{i}^{Q} ,KW_{i}^{K} ,VW_{i}^{V} ) \hfill \\ \end{gathered}$$

The multi-head self-attention on the windows was calculated using the query (Q), key (K), and value (V). During this process, the learnable relative position was used to encode B; this feature can be expressed by Eq. (3) as follows:

4 $${\text{Attention}}\,\,\left( {\text{Q,K,V}} \right) = {\text{SoftMax(QK}}^{{\text{T}}} {/}\sqrt {\text{d}} + {\text{B)V}}$$

For the Swin Transformer, this multi-head self-attention feature was computed n times (the number of Swin Transformer Blocks used in different stages in the network) and then concatenated to obtain the final multi-head self-attention results.

### Swin MSI model for novel mosquito classification

In the real environment, the need to identify novel mosquitoes is common and inevitable. Mosquito species not included in our dataset (which includes 17 species 3 subspecies) are defined herein as novel species. When mosquito images were successfully analyzed by the Swin MSI model, a matrix of size [m, n] was output as C, where m is the number of mosquito images and n is the number of categories used in the process. At this point, the row vector was softmax-normalized to obtain the confidence level of each category in the target image, and this information was used for the species categorization process. By determining the mosquito images in which the mosquito species was accurately identified in this work, the minimum confidence level could be checked and established as the confidence threshold required for novel species detection; if the highest species confidence level of the image to be identified is greater than this threshold, species-level information can be output; if the highest species confidence level is below the threshold, genus-level information can be obtained.

### Identification of morphological keys used by the Swin MSI model

Using gradient-weighted class activation mapping (Grad-CAM)^43^, we visualized and identified the regions in each image that could explain the final classification results obtained by the Swin MSI model. First, the network was forward-propagated to obtain feature layer A (in this work, “feature layer A” refers to the output in each stage) and the network prediction y. Assuming that the categorical prediction of the network for a given mosquito picture is y_c_, the backward-propagation step could provide gradient information A', which was then back-propagated to feature layer A. By calculating the importance of each channel in feature layer A, weighting the sum, and performing the rectified linear unit (ReLu) calculation, the final result was the Grad-CAM. The regions of interest obtained in the attention visualization step were then compared with the morphological classification features that are of interest to mosquito taxonomists to explore whether the Swin Transformer model used similar morphological keys as those used by human experts to classify mosquito species.

### Evaluation methodology

We reported accuracy (acc) and f1-score(f1) in each experiments, the acc can be define as:

$${\text{acc}} = \frac{{{\text{TP}} + {\text{TN}}}}{{{\text{TP}} + {\text{TN}} + {\text{FP}} + {\text{FN}}}}$$

The F1 can be formulated as:

$${\text{f}}1 = \frac{{2 \times {\text{precision}} \times {\text{recall}}}}{{{\text{precision}} + {\text{recall}}}}$$

where precision and recall can be defined as:

$${\text{Precision}} = \frac{TP}{{TP + FP}}$$

$${\text{Recall}} = \frac{TP}{{TP + FN}}$$

where TP is the number of true positive samples, TN is the number of true negative samples, FP is the number of false positive samples and FN is the number of false negative samples.

## Data Availability

The datasets used and analysed during the current study available from the corresponding author on reasonable request.

## Code availability

We have leveraged Github repository for Mask R-CNN, DenseNet, YOLOv5, Detection Transformer, Vision Transformer, and Swin Transformer implementation. The code for Swin MSi are publicly available through GitHub repository: https://github.com/zdz0086/Swin-msi. All are open source and publicly available.
